# Identification of critical genes associated with radiotherapy resistance in cervical cancer by bioinformatics

**DOI:** 10.3389/fonc.2022.967386

**Published:** 2022-07-29

**Authors:** Zhenhua Zhang, Kechao Xiang, Longjing Tan, Xiuju Du, Huailin He, Dan Li, Li Li, Qinglian Wen

**Affiliations:** ^1^ The Affiliated Hospital of Southwest Medical University, Luzhou, China; ^2^ Guangxi Medical University Cancer Hospital, Nanning, China

**Keywords:** bioinformatics, cervical squamous cell carcinoma, radiotherapy, DEGs, prognosis

## Abstract

**Background:**

Cervical cancer (CC) is one of the common malignant tumors in women, Currently, 30% of patients with intermediate to advanced squamous cervical cancer are still uncontrolled or recurrent after standard radical simultaneous radiotherapy; therefore, the search for critical genes affecting the sensitivity of radiotherapy may lead to new strategies for treatment.

**Methods:**

Firstly, differentially expressed genes (DEGs) between radiotherapy-sensitivity and radiotherapy-resistance were identified by GEO2R from the gene expression omnibus (GEO) website, and prognosis-related genes for cervical cancer were obtained from the HPA database. Subsequently, the DAVID database analyzed gene ontology (GO). Meanwhile, the protein-protein interaction network was constructed by STRING; By online analysis of DEGs, prognostic genes, and CCDB data that are associated with cervical cancer formation through the OncoLnc database, we aim to search for the key DEGs associated with CC, Finally, the key gene(s) was further validated by immunohistochemistry.

**Result:**

298 differentially expressed genes, 712 genes associated with prognosis, and 509 genes related to cervical cancer formation were found. The results of gene function analysis showed that DEGs were mainly significant in functional pathways such as variable shear and energy metabolism. By further verification, two genes, ASPH and NKAPP1 were identified through validation as genes that affect both sensitivities to radiotherapy and survival finally. Then, immunohistochemical results showed that the ASPH gene was highly expressed in the radiotherapy-resistant group and had lower Overall survival (OS) and Progression-free survival (PFS).

**Conclusion:**

This study aims to better understand the characteristics of cervical cancer radiation therapy resistance-related genes through bioinformatics and provide further research ideas for finding new mechanisms and potential therapeutic targets related to cervical cancer radiation therapy.

## Introduction

Cervical cancer is one of the most common cancers and a leading cause of cancer death in women (1). The most common pathological type of cervical cancer is squamous cell carcinoma (2). Although cervical cancer can be cured by radical surgery or radiotherapy with equal effectiveness, pelvic chemo-radiation represents the standard therapy for treating locally advanced diseases (3). Concurrent radiotherapy is the most effective treatment for intermediate and advanced stages patients. However, recurrence after radiotherapy remains a problem in treating locally advanced cervical cancer (4). It is clinically essential to find the critical molecular biological mechanisms affecting the intrinsic sensitivity of tumor cells, which can further carry out molecular typing, develop new molecular targeting drugs, and guide and improve the therapeutic efficacy. The molecular mechanisms associated with radiotherapy sensitivity in cervical cancer are very complex, such as the BCL2 family proteins BCL2 and BCL-XL (5), genes such as EGFR, HER-2, p53, p21, Ki-67, HIF, VEGF, COX-2 (6), FA/BRCA pathway of which FANCD2, RAD51, BRCA1, and BRIP1 and other genes (7), but other literature reports different genes and pathways resistant to radiotherapy, the variability of the foothold of each study, the different results obtained, may not be comprehensive, how to analyze these massive gene-related data to get clinically valuable genetic information, bioinformatics method provides us with the research method.

The intrinsic sensitivity of tumor cells to radiotherapy is related to the inherent sensitivity of cells before radiotherapy and the damage repair of tumor cells after radiotherapy, etc. The intrinsic sensitivity of tumor cells results from the joint action of some oncogenes and oncogenes in tumor development, and the damage occurs after tumor cells receive radiotherapy. In contrast, some genetic changes occur damage repair, complex multi-genes, and multiple genetic pathways cross each other in the whole process. The whole process is a complex biological process with numerous genes and multiple genetic pathways intersecting. It is challenging to analyze the radiosensitivity of tumors by analyzing the expression of one or several genes alone in a comprehensive manner. Later molecular biology techniques such as cDNA and gene microarrays were adopted to obtain informative, reproducible, and easy and reliable genetic information and applied to radiotherapy sensitivity studies in cervical cancer (7–9).

Gene expression data analysis is an integral part of bioinformatics and is a hot spot and focus of current bioinformatics research. It reflects the abundance of mRNA, the gene transcription product, in the cell obtained by direct or indirect measurement, which is the data that allows analysis of which genes have been altered in expression, how they are related to each other, and how their activities are affected under different conditions.

## Materials and methods

### Microarray data

We downloaded two CC GEO (10) datasets, including GSE56303 (11) and GSE56363 (7), from the Gene Expression Omnibus (http://www.ncbi.nlm.nih.gov/gds). **Table 1** illustrates the details of GEO cervical cancer data.

**Table 1:** Disease status of 2 sets of genome-wide expression data sets related to radiotherapy and chemotherapy sensitivity of cervical cancer.

GEO serial number	Contributor	Chip platform	Sensitive group (number)	Resistance group (number)
GSE56303	Fernandez-Retana J	NimbleGen	63	22
GSE56363	Balacescu O	Agilent-014850	12	9

### Data normalization and exploration

We first downloaded a series of matrix files, and the platform was converted using Perl programming and scripting language software. Next, the IDs corresponding to the probe names was converted to the international standard names of the genes and then merged into the CSV files. Subsequently, we eliminated the batch effect using the BatchQC (12) package of Bioconductor (http://www.bioconductor.org/). Consequently, we selected the differentially expressed genes (DEGs) using the limma package. Lastly, we selected DEGs, using cutoff criteria of adj.P.Val <0.05 and |logFC | >1 (|logFC| stands for the absolute value of the log fold change and FDR stands for false discovery rate).

### Protein-protein interaction network

We analyzed the PPI pairs of the screened DEGs using the online database STRING (13) version 11 (https://string-db.org/). Next, we constructed to PPI network of DEGs and selected one interaction that was statistically significant with a composite score >0.4.

### GO enrichment analyses of DEGs

To identify DEGs associated pathways and function annotations, Gene Ontology (GO) was conducted by DAVID (9) online database (DAVID; https://david.ncifcrf.gov). GO is a widely used ontology in the field of bioinformatics, which covers three aspects of biology: biological process (BP), cellular component (CC), and molecular function (MF) (14). P-value < 0.05 indicated statistically significant difference.

Validation of DEGs by the HPA, CCDB, and OncoLnc Database

First, the survival-associated genes of cervical cancer were downloaded from the Human Protein Atlas (HPA)database (15), (http://www.proteinatlas.org/humanproteome/pathology) which contains transcriptome-wide data on protein-coding genes associated with clinical outcomes in 17 major cancers and provides genes associated with survival in each cancer. Next, genes associated with the formation process of cervical cancer were downloaded from Cervical Cancer Gene Database (CCDB) (16) (http://crdd.osdd.net/raghava/ccdb). CCDB is a manually compiled experimentally validated catalog containing genes involved in different stages of the cervical cancer formation process. Subsequently, Intersections were taken between DEGs and genes obtained from the HPA databases and CCDB, using the Venn R package. Finally, we performed overall survival (OS) analysis of intersecting genes using the OncoLnc database (http://www.oncolnc.org/), which contains survival data from 8647 patients with 21 tumors in TCGA and provides an online survival analysis.

### Tissue collection

From January 2011 to June 2015 normal cervical, intermediate, and advanced squamous cervical cancer tissue samples were acquired from the Department of Pathology, Affiliated Cancer Hospital of Guangxi Medical University. All specimens were subjected to immunohistochemical evaluation and confirmed by two independent pathologists. This study was approved by the research and clinical trial ethics committee of Affiliated Cancer Hospital of Guangxi Medical University, and all eligible participants provided written informed consent. All clinical procedures were performed per the ethical standards of the Declaration of Helsinki guidelines and relevant policies in China.

### Immunohistochemistry

**Figure 1:**
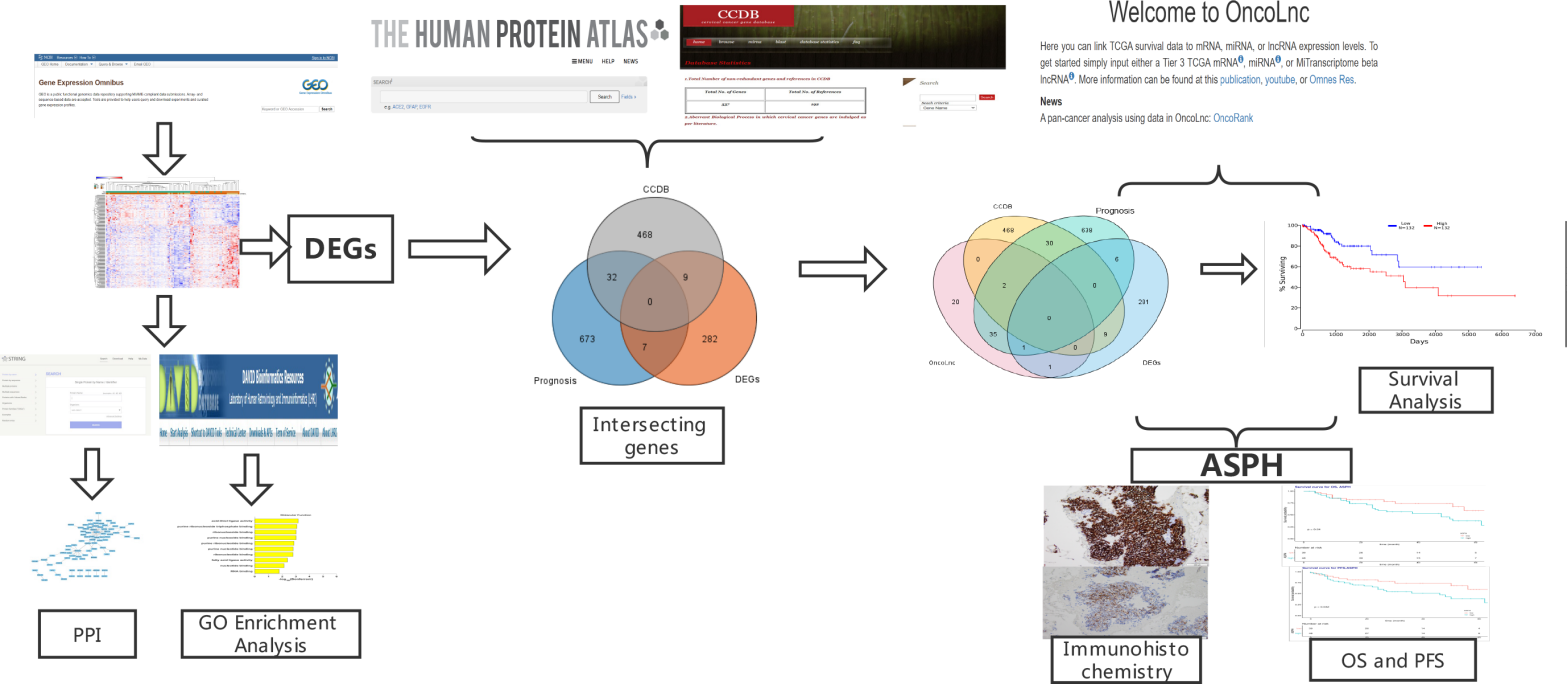
Flow Diagram.

To validate Kinetochore Aspartate Beta-Hydroxylase (ASPH), immunohistochemical staining was performed on the specimen sections of radiotherapy sensitivity and resistance samples. The following steps were followed: section dewaxing, antigen repair, ASPH antibody (Bio-Swamp Company) incubation, and secondary antibody incubation. The flow chart is shown in **Figure 1**.

### Statistical analysis

All data were analyzed using R language 3.4.3 software (https://CRAN.R-project.org/package=survival.), survival curves and survival rates were plotted using the Kaplan-Meier method, and differences in survival rates between groups were tested using the log-rank test (log-rank method), and P-values were obtained using the chi-square test. Univariate and multifactorial analyses were performed using the ratio COX regression model with 95% confidence intervals (95% CI) for the risk ratio area, and all data were analyzed using a two-sided test, with P<0.05 indicating statistical differences.

## Results

### Batch effect treatment results

**Figure 2:**
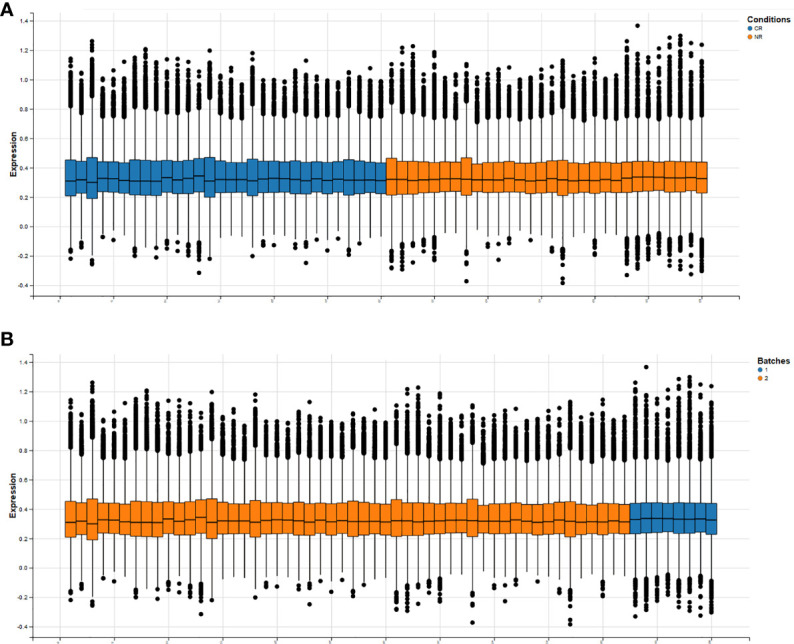
Overall expression levels of individual samples after removal of batch effects (horizontal axis is all samples, vertical axis is normalized expression values. **(A)** shows the expression information of genes in each sample under both conditions, **(B)** shows the expression information of genes in each sample under both batches.).

**Figure 3:**
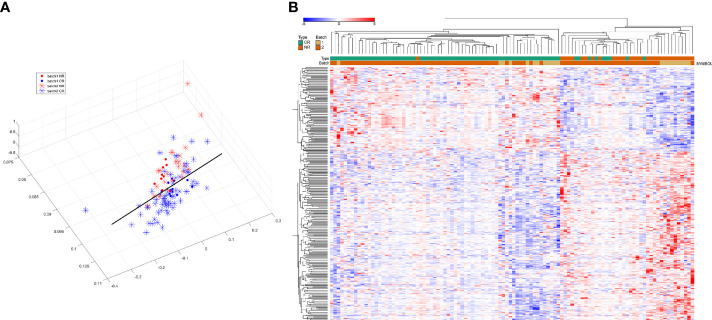
**(A)** Results of principal component analysis (red and blue for distinguishing between batches, asterisks and solid circles for distinguishing between sensitive and control groups). **(B)**: hierarchical clustering analysis of 298 differentially expressed genes.

The BatchQC package was used to remove the batch effects from the two combined expression data sets, and the results showed that the overall expression levels were similar between the samples in **Figure 2**. On the one hand, to test the effect of removing the batch effect, and on the other hand, to test whether the batch effect could distinguish the radiotherapy-sensitive group from the radiotherapy-resistant group, a principal component analysis (PCA) was done on the data after removing the batch effect. The first three were taken for the PCA in **Figure 3A**. the figure below that the batch effect is not entirely removed. However, the results are still acceptable, and at the same time, the two groups are relatively well differentiated.

### Identification of DEGs

After removing the batch effect, the data were combined and analyzed together, with batch as a covariate, and using the limma package, 298 differentially expressed genes (DEGs) were calculated, of which 199 were up-regulated and 99 were down-regulated. Then we used these differentially expressed genes for sample clustering. The 298 differentially expressed genes can be ideally distinguished between the radiotherapy-sensitive and resistant groups. The results are shown in **Figure 3B**.

### Gene function analysis and PPI network construction

**Figure 4:**
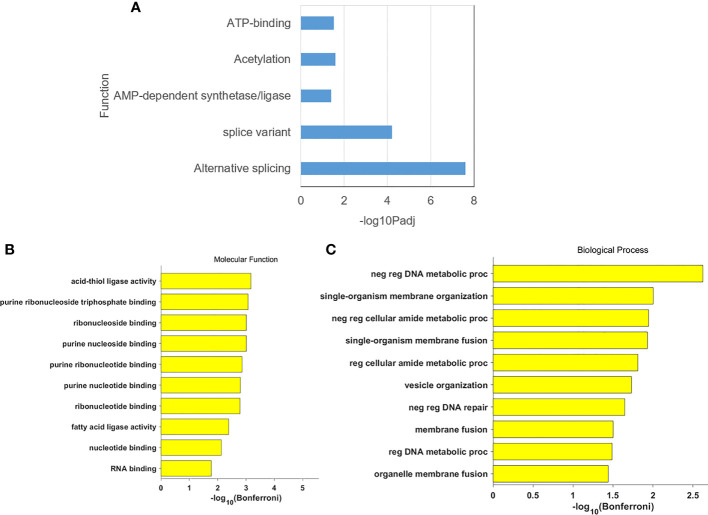
**(A)** The gene annotation in DEGs; **(B)** The cluster result of molecular function in DEGs: **(C)** The cluster result of biological process in DEGs.

Enrichment analysis of DEGs for functional classification showed that DEGs were essential mainly in functional pathways such as variable shear and energy metabolism in **Figure 4A**. GO functional enrichment showed that DEGs are mainly associated with base binding related to molecular functions, and DNA stability functions, cell, and organ cell membranes in biological processes in **Figures 4B**, **C**.

**Figure 5:**
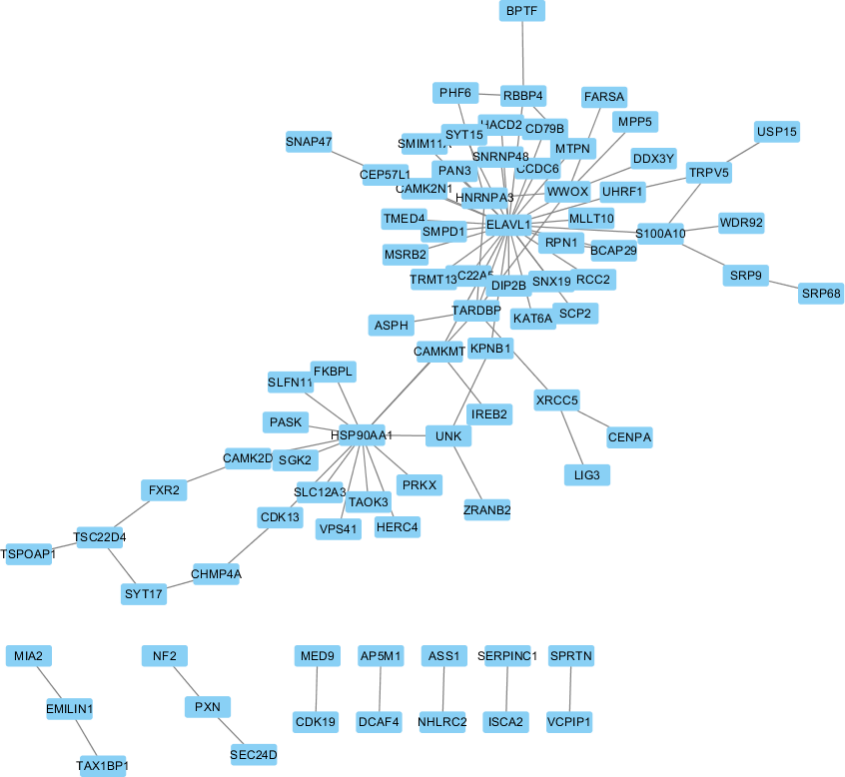
Protein-Protein interactions between DEGs.

We downloaded all human protein-protein interaction pairs from STRING, and based on this network, all protein-protein interactions between DEGs will be extracted. The results are shown in **Figure 5**. theater network is the ELAVL1 gene, which encodes the ELAV1 protein. This RNA-binding protein is involved in the differentiation of embryonic stem cells and is associated with the prognosis of various cancers. This is followed by the HSP90AA1 gene, which encodes a protein primarily involved in ATPase activity.

### Validation of DEGs

**Figure 6:**
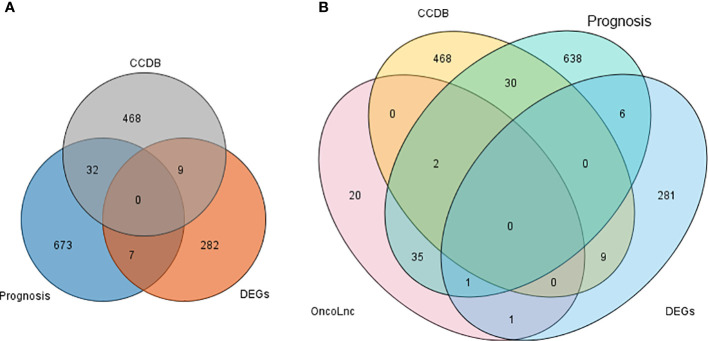
**(A)** Venn diagram ofthree gene database sources. **(B)** Venn diagram of three gene database sources with OncoLnc.

First, validation was performed on CCDB and HPA databases, and Venn diagrams were obtained for DEGs, 712 prognosis genes (referred to as Prognosis) from the HPA database, and 509 genes associated with cervical cancer formation from CCDB (referred to as CCDB data) in this study. The results showed no genes were found that intersected in all 3 groups. While seven genes with the intersection of DEGs and Prognosis were found, nine genes with the intersection of DEGs and CCDB data are shown in **Figure 6A** and **Table 2**.

**Table 2:** Genes at the intersection of 3 databases.

DEGs & Prognosis	DEGs & CCDB	Prognosis & CCDB		
ASPH DENND2D ETFB FARSA HNRNPA3 RCC2 TARDBP	ATP9A CXCR2 FGF1 GTF2F2 HSP90AA1 LSM3 S100A10 S100P TWIST1	ADAM9 AQP3 BCL2 CDA DSG2 E2F1 EREG FGFR2 GLTP IL1A ITGB1	LDHA MCM2 MCM3 MCM5 MDM2 MGMT MMP1 MMP3 NME2 OSMR PCNA	PDK2 PLOD2 POP5 PTPN6 RARRES3 RASSF1 SPP1 TFRC TP73 VEGFA

**Figure 7:**
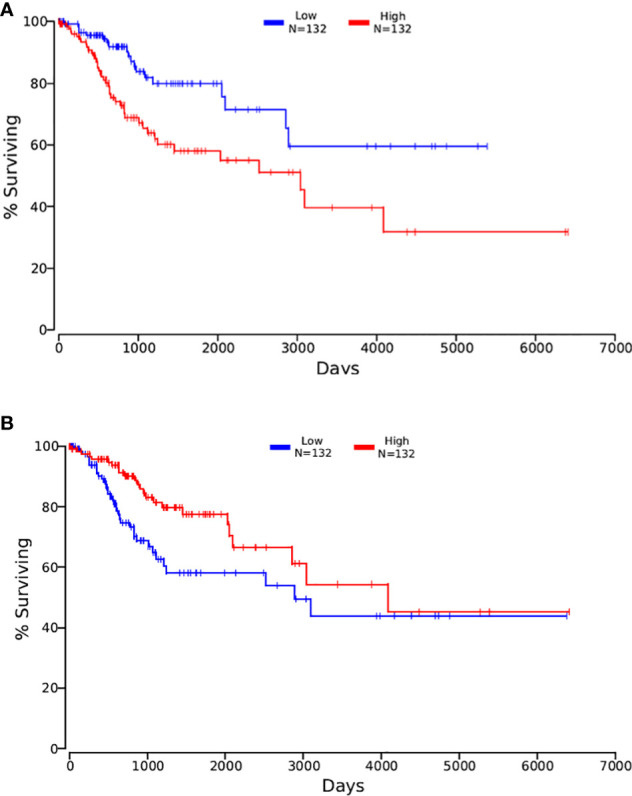
**(A)** the Kaplan-Meirer plot of ASPH in OncoLnc; **(B)** the Kaplan-Meirer plot of NKAPPl in OncoLnc.

Then, the 3 data from the previous period were intersected with OncoLnc. The results are shown in **Figure 6B**. After the clinical survival validation for each gene, we analyzed with 50 as a lower and upper percentile. There was no difference in the analysis of survival curves for any of the nine genes obtained by intersecting DEGs with CCDB. Validating the acquired DEGs with the seven genes that had an intersection with the Prognosis group, only ASPH and NKAPP1 had differences in survival curves with a statistically significant P<0.05, as shown in **Figures 7A**, **B**. From survival curves, it can be seen that the low expression of ASPH and high expression of NKAPP1 may lead to increased sensitivity to radiotherapy, thus improving cervical squamous cancer patients’ efficacy.

### Immunohistochemical results

#### ASPH expression results and patient’s general condition

To further validate the relationship between ASPH and sensitivity to radiotherapy, we performed immunohistochemistry. We finally obtained 87 specimens from the Affiliated Cancer Hospital of Guangxi Medical University, including 44 cases in the radiotherapy-sensitive group and 43 cases in the radiotherapy-resistant group. All 87 patients who underwent ASPH expression analysis, combined with the retrospective data analysis, their basic information is shown in **Table 3**. High expression of ASPH in the radiotherapy-resistant group accounted for 69.76% (30/43). High expression in the radiotherapy-sensitive group accounted for 40.9% (18/26), the rate of increased expression of ASPH in the radiotherapy-resistant group was higher than that in the radiotherapy-sensitive group, p< 0.05, and the difference was statistically significant grouped according to ASPH expression, between the two groups, the mortality rate of those with high ASPH expression was 54.2% significantly higher than that of 28.2% in the low expression group and statistically different, and the difference between their OS and PFS was not statistically significant. There were no significant differences in age, stage, tumor diameter, lymph node metastasis, NLR, PLR, lymph node metastasis, total time of radiotherapy, number of concurrent chemotherapy sessions (TR), and number of adjuvant chemotherapy sessions between the two groups.

**Table 3:** General characteristics of the patient.

Clinical factors	ASPH low expression group (N=39)	ASPH high expression group (N=48)	P-value
Radiochemoradiotherapy sensitivity (number, %)			0.013
Sensitivity to chemoradiotherapy	26 (66.7)	18 (37.5)	
Chemoradiotherapy resistance	13 (33.3)	30 (62.5)	
Age (years)	54.03 (10.36)	53.69 (7.90)	0.863
Survival status number, %)			0.027
Survival	28 (71.8)	22 (45.8)	
Dead	11 (28.2)	26 (54.2)	
OS (mouth)	32.82 (19.62)	30.65 (19.88)	0.611
PFS (mouth)	31.44 (20.54)	27.54 (20.39)	0.380
stage(number, %)			0.385
IIb-IIIa	14 (35.9)	12 (25.0)	
≥IIIb	25 (64.1)	36 (75.0)	
Tumor diameter (number, %)			0.551
<=4cm	18 (46.2)	18 (37.5)	
>4cm	21 (53.8)	30 (62.5)	
hemoglobin (mean (SD))	106.51 (22.71)	110.29 (21.82)	0.432
NLR (mean (SD))	3.01 (1.82)	3.15 (1.66)	0.715
PLR (mean (SD))	187.85 (116.10)	186.15 (82.97)	0.937
Lymph node metastasis number, %)			0.722
No	22 (56.4)	30 (62.5)	
Yes	17 (43.6)	18 (37.5)	
Total radiotherapy time (%)			0.659
TR<= eight week	19 (48.7)	20 (41.7)	
TR > 8 week	20 (51.3)	28 (58.3)	
Number of concurrent chemotherapy courses (times)	3.03 (1.77)	3.60 (1.54)	0.107
Number of adjuvant chemotherapy courses (times)	1.38 (1.55)	1.38 (1.57)	0.977

#### ASPH expression profile and survival analysis results

**Figure 8:**
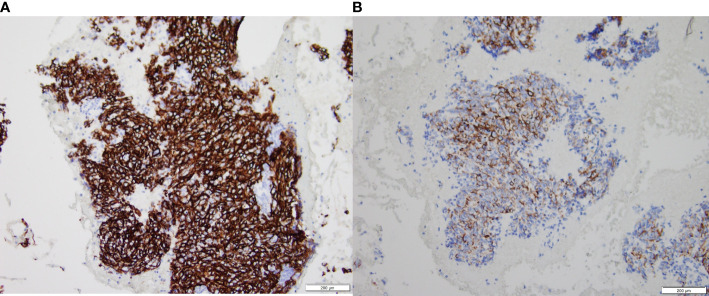
ASPH expression in intermediate and advanced cervical cancer tissues, **(A)** is high expression results. **(B)** is low expression results.

At the end of the follow-up, 50 of the 87 patients were alive. There were significant differences in OS, PFS, and survival between the ASPH low expression group and the high expression group, and significantly lower OS and PFS in those with high ASPH expression, and significant differences. **Figure 8** shows the expression of ASPH in cervical cancer tissues.

### Analysis of clinical factors affecting OS and PFS of CC

**Figure 9:**
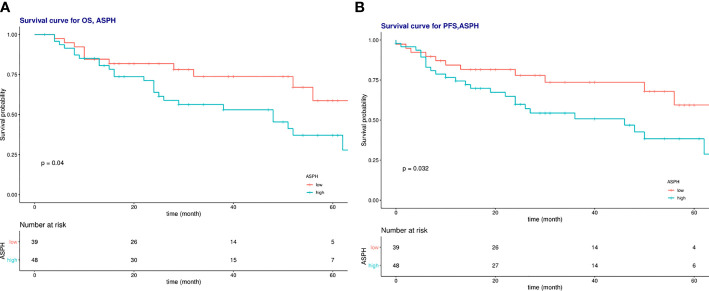
**(A)** Survival curves of overall survival in low ASPH expression and high ASPH expression; **(B)** Survival c\llVes of progression-free survival in low ASPH expression and high ASPH expression.

Univariate analysis showed that high ASPH expression was a prognostic indicator of OS, with 2.068 times the risk of high ASPH expression compared with low expression (HR=2.06, 95% CI=1.02-4.18, P<0.005) in **Figure 9A**. However, the multifactorial analysis did not show statistical differences. Among other factors, univariate analysis of radiotherapy sensitivity, tumor diameter, NLR, lymph node metastasis status, and total duration of radiotherapy were influential factors of OS, and multifactorial analysis showed radiotherapy sensitivity, tumor diameter, etc. and the entire course of radiotherapy were independent factors affecting OS (See **Table 4**).

**Table 4:** Analysis of clinical factors affecting OS of radiotherapy and chemotherapy for intermediate and advanced cervical squamous cell carcinoma.

Clinical factors	cases	Univariate analysis		Multivariate analysis	
		HR(95% CI)	P-value	HR (95% CI)	P-value
group			<0.001		<0.001
Chemoradiotherapy sensitive group	44	ref		ref	
Chemoradiotherapy resistance group	43	4.44 (2.09-9.43)		5.67 (2.37-13.56)	
ASPH			0.0448		0.2602
low	39	ref		ref	
high	48	2.06 (1.02-4.18)		1.60 (0.71-3.62)	
age(year)	87	0.99 (0.95-1.03)	0.564	0.97 (0.92- 1.03)	0.3411
Stage			0.243		0.3155
IIb-IIIa	26	ref		ref	
≥IIIb	61	1.60 (0.73-3.53)		1.60 (0.64-4.04)	
Tumor diameter			0.0161		0.0735
<=4cm	36	ref		ref	
>4cm	51	2.33 (1.12-4.81)	0.0229	3.22 (1.29-8.04)	0.0124
hemoglobin	87	1.00 (0.98-1.01)	0.776	1.01 (0.99-1.03)	0.2566
NLR	87	1.27 (1.06-1.52)	0.0086	1.02 (0.79-1.31)	0.9067
PLR	87	1.00 (1.00-1.00)	0.203	1.00 (1.00-1.00)	0.8209
Lymph node metastasis			0.018		0.3182
No	52	ref		ref	
Yes	35	2.20 (1.15-4.22)		1.51 (0.67-3.38)	
The total duration of radiotherapy (month)					
<=8	39	ref		ref	
> 8	48	2.06 (1.03-4.12)	0.0411	2.82 (1.24-6.40)	0.0132
Number of concurrent chemotherapy courses (weeks)	87	0.94 (0.79-1.12)	0.469	0.82 (0.64-1.04)	0.1052
Adjuvant chemotherapy course (weeks)	87	0.99 (0.80-1.23)	0.946	0.97 (0.74-1.26)	0.7997

Univariate analysis showed that high ASPH expression was a prognostic indicator of PFS, with a 4.8-fold higher risk for high ASPH expression than for low expression (HR=2.12, 95% CI=1.05-4.30, P<0.005) in **Figure 9B**. However, the multifactorial analysis did not show statistical differences. Among other factors, univariate analysis of radiotherapy sensitivity, tumor diameter, NLR, lymph node metastasis status, and total duration of radiotherapy were influential factors of PFS. In contrast, the multifactorial analysis showed that radiotherapy sensitivity, tumor diameter, and the time of radiotherapy were independent factors affecting PFS (See **Table 5**).

**Table 5:** Analysis of clinical factors affecting PFS of radiotherapy and chemotherapy in intermediate and advanced cervical squamous cell carcinoma.

Clinical factors	Cases	Univariate analysis		Multivariate analysis	
		HR (95% CI)	P-value	HR (95% CI)	P-value
group			<0.001		<0.001
Chemoradiotherapy sensitive group	44	ref		ref	
Chemoradiotherapy resistance	43	4.80 (2.26-10.20)		6.10 (2.58-14.41)	
ASPH			0.0369		0.3326
low	39	ref		ref	
high	48	2.12 (1.05-4.30)		1.50 (0.66-3.39)	
Age	87	0.99 (0.95-1.03)	0.536	0.97 (0.92-1.03)	0.2989
Stage					
IIb-IIIa	26	ref		ref	
≥IIIb	61	1.65 (0.75-3.62)	0.216	1.60 (0.63-4.04)	0.3250
Tumor diameter					
<=4cm	36	ref		ref	
>4cm	51	2.35 (1.14-4.87)	0.0213	3.33 (1.31-8.45)	0.0115
hemoglobin	87	1.00 (0.98-1.01)	0.758	1.01 (0.99-1.03)	0.2463
NLR	87	1.25 (1.05-1.50)	0.0132	0.99 (0.78-1.27)	0.9595
PLR	87	1.00 (1.00-1.01)	0.186	1.00 (1.00-1.01)	0.7840
Lymph node metastasis			0.0194		0.3037
No	52	ref		ref	
Yes	35	2.18 (1.13-4.18)		1.52 (0.69-3.37)	
The total duration of radiotherapy (month)					
<=8	39	ref		ref	
> 8	48	2.04 (1.02-4.08)	0.0433	2.54 (1.14-5.66)	0.0231
Number of concurrent chemotherapy courses (weeks)	87	0.93 (0.78-1.12)	0.453	0.80 (0.63-1.02)	0.0667
Adjuvant chemotherapy course (weeks)	87	1.01 (0.81-1.25)	0.956	0.99 (0.76-1.28)	0.9193

## Discussion

In addition to the clinicopathologic-related factors, the efficacy of radiotherapy for cervical cancer is related to the intrinsic sensitivity of tumor cells to radiotherapy, and the inherent resistance to radiotherapy is a critical factor that needs to be unresolved, which is also the focus and hot spot of research in recent years. In 2005 Gaffney, D. K (17). reported that gene expression analysis using RNA amplification after pathological tissue biopsy from patients with cervical cancer was feasible, laying the foundation for future studies. Weidhaas, J.B (18). found that gene expression was altered during radiotherapy for cervical cancer, predicting treatment efficacy. Later many researchers started to use this technique to perform analysis of gene expression in radiotherapy-sensitive and resistant groups with the expectation of finding genes and pathways associated with radiotherapy resistance to guide treatment to improve prognosis.

The current published studies using gene microarray studies regarding the analysis of radiotherapy sensitivity in cervical cancer retrieved from PubMed are An, J.S (19) at the Cancer Hospital of Chinese Academy of Medical Sciences in 2013 at the earliest (12), Balacescu, O (7) in 2014 and Fernandez-Retana, J (11) in 2015, and these three papers, the first one failed to include the relevant data in this bioinformatics analysis because it did not upload the relevant expressed genes to public databases such as GEO. Only the latter two were included in this study for bioinformatics analysis. We used bioinformatics to obtain differentially expressed genes for these 2 data sets and analyzed them in functional gene classification, molecular function, biological processes, and protein interactions. The results showed that the differentially expressed genes obtained could well distinguish radiotherapy-sensitive from radiotherapy-resistant, in terms of gene functional classification mainly in the available pathways, especially in variable shear and energy metabolism, and found that the proteins involved in The two main ELAVL1 and HSP90AA1 genes involved in protein interactions were identified. The results are somewhat different from the literature published by An, J.S et al. This study showed that radiotherapy sensitivity was related to multiple pathways: DNA damage repair, apoptosis, cell cycle, MAPK signaling pathway, anaerobic glycolysis, and glutathione metabolism, in the radiotherapy-sensitive group SMUG1 and CDK7 genes, were downregulated in the DNA damage repair pathway, ATM genes were upregulated, in the MAPK pathway IL1R1, PDGFRA and TGFB3 genes upregulated and HRAS downregulated, IL1R1, PRKAR1A, and ATM upregulated while CAST, BNIP3, and BAK1 downregulated in tumor necrosis-related genes, and TGFB3 and ATM upregulated while CDK7, RAN and HRAS downregulated in cell cycle-related genes. The same study by Balacescu O (7), also included in our bioinformatics analysis, considered the DNA damage repair pathway as a significant pathway study for radiotherapy sensitivity, which showed differences in 17 genes on this pathway (RAD51, BRIP1, BLM, BRCA1, BRCA2, BRCC3, HLTF, FANCD2 FANCI, FANCM, FANCL, ATF1, E2F4, E2F2, SMARCA2, SMARCA4, and RFC1), with a particular focus on which overexpression of BRCA1, BRCA2, RAD51, BRIP1 (BACH1), FANCD2, BLM, and RFC in the radiotherapy-resistant group can be detected in their pathways by cell cycle arrest and homologous sufficiency leading to the activation of DNA repair mechanisms. In addition, Kitahara, O (9), studying the expression of radiotherapy sensitivity genes in cervical cancer with radiotherapy alone, found 171 genes differentially between the radiotherapy sensitive and resistant groups, 121 genes were upregulated, and 50 genes were downregulated in the keen radiotherapy group. Upregulation of genes related to the MAPK pathway such as MAP3K2 and RAB5C family in the gene pathway played an important role in radiotherapy. The upregulation of MAPK pathway-related genes such as MAP3K2 and RAB5C family played an important role in radiotherapy sensitivity. The downregulation of DNA repair-related genes such as XRCC5 in the radiotherapy sensitive group and the downregulation of other pathway genes such as LDHA mainly focused on the high expression of XRCC5 in the radiotherapy resistant and the increased expression of ALDH1 and RBP1 in the radiotherapy sensitive group. A J.S et al. found that the PDGFRA and PRKAR gene families were consistently upregulated in the radiotherapy-sensitive group. Still, no overlapping consistent genes were found between these publications and the results of the current bioinformatics analysis.

Most of the above studies found partial overlap with radiotherapy-sensitive related gene pathways—still, fewer studies on whether radiotherapy-sensitive genes are directly associated with prognosis. We found seven genes consistent with forecast and nine genes consistent with the formation process of cervical cancer by bioinformatics. However, we did not find genes common to the 3 data sets. We found differentially expressed genes affecting radiotherapy sensitivity in cervical cancer by gene function classification and analysis with proteomic data showed that the primary function is on the pathway of energy metabolism, which is different from the path affecting prognosis differently, suggesting that the two may work together through synergy to accomplish prognosis-related effects. We identified two genes, ASPH and NKAPP1, as genes that affect radiotherapy sensitivity and survival through experimental and clinically relevant data validation.

The NKAPP1 (NFKB activating protein pseudogene) gene is an NK-κB activating protein pseudogene, a segment of the DNA base sequence that is very similar to the line of a gene that has been in other organisms but is unable to perform its original function and makes proteins. Still, studies have shown that these genes are often involved in transcriptional regulation and play a role in multiple positions in cancer pathogenesis. Cancer subtype analysis can be used as a prognosis-related biological indicator (20, 21). Their expression in pathological tissue specimens could not be studied in this study because of the absence of protein function.

ASPH gene (aspartate beta-hydroxylase), aspartate-aspartate beta-hydroxylase is a highly conserved deoxygenase present in cells since the embryonic stage in mammalian arteries (22).ASPH gene DNA length is 2277bp and contains 27 exons, which encodes 4 of the proteins ASPH, HUMBUG Junctate, and junction, of which ASPH is the primary translation product. ASPH overexpression may promote tumor cell formation, proliferation, invasion, and metastasis (22), becoming an indicator of malignant expression and considered a potential tumor marker, which is currently more studied in hepatocellular carcinoma. The positive expression rate of ASPH in cervical cancer cells is about 88.5% (23). There are no relevant studies on its role in cervical cancer and related functional studies on whether there is a difference in ASPH expression in radiotherapy-sensitive and resistant groups.

The final results showed that in intermediate and advanced cervical squamous carcinoma specimen pathology, the high expression of ASPH was higher in the radiotherapy-resistant group than in the radiotherapy-sensitive group. The increased expression of ASPH affected the OS and PFS of patients, and those with high expression had significantly lower OS and PFS than those with a common word. The univariate analysis of ASPH high expression showed that ASPH was one of the prognostic influencing factors affecting OS and PFS. Still, the multifactorial analysis did not yield consistent results, which may be related to the small sample size, with only 87 cases included in the research. Whether increasing the number of patients may yield positive results, which needs to be confirmed by more studies. There are very few studies on ASPH in cervical cancer, one published *in vitro* study showed that ASPH was positively expressed in 3 specimens in cervical cancer cell lines. Still, the results were not very convincing because of the *in vitro* experiment and the very effective sample size. ASPH in cervical cancer and radiotherapy-related studies is even less reported (24).

ASPH may become a new tumor marker. Since most studies have shown that ASPH is overexpressed in malignant tumor cells and low or no expression in normal tissues and overexpressed ASPH can be detected by releasing from tumor cells into human serum and body fluids, ASPH can be used as a novel tumor marker and therapeutic target. Jizong Zhang (25) showed that ASPH alone has lower sensitivity than GP73 but higher than AFP for detecting primary liver cancer, but lower specificity than both 2. However, ASPH combined with AFP and GP73 is higher than the single test, with 96% sensitivity, 98% specificity, and 97% accuracy. Whether both can be used as tumor markers for other tumors, including cervical cancer, needs to be explored in more studies.

ASPH may become a marker suggestive of prognosis. Studies of primary liver cancer showed (26) that hepatocellular carcinoma cells with high expression of ASPH are more active, migratory, invasive, and metastatic, and et al. similarly confirmed (27) that ASPH could promote cancer cell re-interrogation migration, *in vivo* metastasis. Distant metastasis through relevant signaling pathways suggests that tumor cells are more aggressive. *In vivo* studies using a computer-assisted synthesis of compounds that inhibit β-hydroxylase have shown to inhibit the North signaling pathway in hepatocellular carcinoma to produce antitumor effects. In cholangiocarcinoma (28), ASPH may promote cholangiocarcinoma progression by regulating RB1 phosphorylation. We showed that high expression of ASPH in cervical cancer is more likely to resist radiotherapy, suggesting that possibly those with high ASPH expression have poor sensitivity to treatment with radiotherapy.

ASPH may also be a new pathway for therapy. Studies in pancreatic cancer (29) showed that the drug SNS-622-DM1 coupled with ASPH has a better antitumor effect *in vitro*. Animal experiments can limit proliferation, promote apoptosis, and significantly inhibit tumor growth and metastatic foci. In the study of hepatocellular carcinoma, the antigenicity of ASPH can be used to load ASPH on dendritic cells to induce the production of CD4+ T cells, and the antitumor effect can be achieved through this immune response (30). Such immunotherapy can also inhibit the cytotoxicity of bile duct cancer cells and suppress the growth and metastasis of intrahepatic tumors.

Our study showed a higher rate of ASPH positive expression in the radiotherapy-resistant group in intermediate to advanced squamous cervical cancer. It is speculated that there may be higher concentrations of ASPH in the blood, and it may be possible that the sensitivity of radiotherapy may be enhanced by corresponding immunotherapy or targeted drugs, thus improving the efficacy. Still, of course, this all needs to be verified by further studies.

## Conclusion

We identified seven genes consistent with prognosis and nine genes consistent with the process of cervical cancer formation, among which ELAVL1 and HSP90AA1 are involved in protein inter-righting. We identified two genes, ASPH and NKAPP1, as both genes affecting radiotherapy sensitivity and survival by analyzing the included GEO database on radiotherapy sensitivity in intermediate and advanced squamous cervical cancer, using bioinformatics methods with relevant databases. Immunohistochemical results showed that ASPH was more highly expressed in the radiotherapy-resistant group than in the radiotherapy-sensitive group in intermediate and advanced squamous cervical cancer. Those with high ASPH expression had lower OS and PFS and could be prognostic indicators in intermediate and advanced squamous cervical cancer. ASPH could be a tumor marker, prognostic indicator, and therapeutic target in squamous cervical cancer.

## Data availability statement

The raw data supporting the conclusions of this article will be made available by the authors, without undue reservation.

## Ethics statement

Written informed consent was obtained from the individual(s) for the publication of any potentially identifiable images or data included in this article.

## Author contributions

LL and QW were the corresponding authors, mainly providing pathological information and subject ideas. ZZ is the first author and is the main implementer of the whole experiment. KX is the co-first author, mainly refining the bioinformatics analysis. LT and HH are primarily responsible for data collection and collation. DL and XD are mainly for bioinformatics analysis and article revision and retouching. All authors contributed to the article and approved the submitted version.

## Conflict of interest

The authors declare that the research was conducted in the absence of any commercial or financial relationships that could be construed as a potential conflict of interest.

## Publisher’s note

All claims expressed in this article are solely those of the authors and do not necessarily represent those of their affiliated organizations, or those of the publisher, the editors and the reviewers. Any product that may be evaluated in this article, or claim that may be made by its manufacturer, is not guaranteed or endorsed by the publisher.

## References

[1] Shrivastava S Mahantshetty U Engineer R Chopra S Hawaldar R Hande V. Cisplatin chemoradiotherapy vs radiotherapy in FIGO stage IIIB squamous cell carcinoma of the uterine cervix. JAMA Oncol (2018) 4:506. doi: 10.1001/jamaoncol.2017.5179 29423520 PMC5885185

[2] Wu S Huang E Lin H. Optimal treatments for cervical adenocarcinoma. Am J Cancer Res (2019) 9:1224–34. PMC6610057 31285954

[3] Santin AD Sill MW McMeekin DS Leitao MM Brown J Sutton GP. Phase II trial of cetuximab in the treatment of persistent or recurrent squamous or non-squamous cell carcinoma of the cervix: A gynecologic oncology group study. Gynecol Oncol (2011) 122:495–500. doi: 10.1016/j.ygyno.2011.05.040 21684583 PMC3152667

[4] Kim MK Kim T Sung C Choi CH Lee J Bae D. Clinical significance of HIF-2α immunostaining area in radioresistant cervical cancer. J Gynecol Oncol (2011) 22:44. doi: 10.3802/jgo.2011.22.1.44 21607095 PMC3097334

[5] Wootipoom V Lekhyananda N Phungrassami T Boonyaphiphat P Thongsuksai P. Prognostic significance of bax, bcl-2, and p53 expressions in cervical squamous cell carcinoma treated by radiotherapy. Gynecol Oncol (2004) 94:636–42. doi: 10.1016/j.ygyno.2004.03.012 15350352

[6] Petera J Sirak I Beranek M Vosmik M Drastikova M Paulikova S. Molecular predictive factors of outcome of radiotherapy in cervical cancer. Neoplasma (2011) 58:469–75. doi: 10.4149/neo_2011_06_469 21895399

[7] Balacescu O Balacescu L Tudoran O Todor N Rus M Buiga R. Gene expression profiling reveals activation of the FA/BRCA pathway in advanced squamous cervical cancer with intrinsic resistance and therapy failure. BMC Cancer (2014) 14:246. doi: 10.1186/1471-2407-14-246 24708616 PMC4021393

[8] Harima Y Togashi A Horikoshi K Imamura M Sougawa M Sawada S. Prediction of outcome of advanced cervical cancer to chemoradiotherapyression profiles of 35 genes selected by cDNA microarray analysis. Int J Radiat Oncol Biol Phys (2004) 60:237–48. doi: 10.1016/j.ijrobp.2004.02.047 15337562

[9] Kitahara OO Katagiri TT Tsunoda TT Harima YY Nakamura YY. Classification of sensitivity or resistance of cervical cancers to ionizing radiation according to expression profiles of 62 genes selected by cDNA microarray analysis1. Neoplasia (New York NY) (2002) 4:295–303. doi: 10.1038/sj.neo.7900251 PMC1531706 12082545

[10] Barrett T Wilhite SE Ledoux P Evangelista C Kim IF Tomashevsky M. NCBI GEO: Archive for functional genomics data sets–update. Nucleic Acids Res (2012) 41:D991–5. doi: 10.1093/nar/gks1193 PMC3531084 23193258

[11] Fernandez-Retana J Lasa-Gonsebatt F Lopez-Urrutia E Coronel-Martínez J Cantu De Leon D Jacobo-Herrera N. Transcript profiling distinguishes complete treatment responders with locally advanced cervical cancer. Transl Oncol (2015) 8:77–84. doi: 10.1016/j.tranon.2015.01.003 25926073 PMC4415118

[12] Manimaran S Selby HM Okrah K Ruberman C Leek JT Quackenbush J. BatchQC: Interactive software for evaluating sample and batch effects in genomic data. Bioinformatics (2016) 32:3836–8. doi: 10.1093/bioinformatics/btw538 PMC5167063 27540268

[13] Szklarczyk D Gable AL Lyon D Junge A Wyder S Huerta-Cepas J. STRING v11: Protein-proteintworks with increased coverage, supporting functional discovery in genome-wide experimental datasets. Nucleic Acids Res (2019) 47:D607–13. doi: 10.1093/nar/gky1131 PMC6323986 30476243

[14] Ashburner M Ball CA Blake JA Botstein D Butler H Cherry JM. Gene ontology: Tool for the unification of biology. Nat Genet (2000) 25:25–9. doi: 10.1038/75556 PMC3037419 10802651

[15] Uhlen M Zhang C Lee S Sjöstedt E Fagerberg L Bidkhori G. A pathology atlas of the human cancer transcriptome. Science (2017) 357(6352):eaan2507. doi: 10.1126/science.aan2507 28818916

[16] Agarwal SM Raghav D Singh H Raghava GPS. CCDB: A curated database of genes involved in cervix cancer. Nucleic Acids Res (2010) 39:D975–9. doi: 10.1093/nar/gkq1024 PMC3013652 21045064

[17] Gaffney DK Winter K Fuhrman C Flinner R Greven K Ryu J. Feasibility of RNA collection for micro-array gene expression analysis in the treatment of cervical carcinoma: A scientific correlate of RTOG c-0128. Gynecol Oncol (2005) 97:607–11. doi: 10.1016/j.ygyno.2005.01.014 15863167

[18] Weidhaas JB Li S Winter K Ryu J Jhingran A Miller B. Changes in gene expression predicting local control in cervical cancer: Results from radiation therapy oncology group 0128. Clin Cancer Res (2009) 15:4199–206. doi: 10.1158/1078-0432.CCR-08-2257 PMC2758917 19509178

[19] An JS Huang MN Song YM Li N Wu LY Zhan QM. A preliminary study of genes related to concomitant chemoradiotherapy resistance n advanced uterine cervical squamous cell carcinoma. Chin Med J (Engl) (2013) 126:4109–15. 24229683

[20] Xiao-Jie L Ai-Mei G Li-Juan J Jiang X. Pseudogene in cancer: Real functions and promising signature. J Med Genet (2014) 52:17–24. doi: 10.1136/jmedgenet-2014-102785 25391452

[21] Han L Yuan Y Zheng S Yang Y Li J Edgerton ME. The pan-cancer analysis of pseudogene expression reveals biologically and clinically relevant tumor subtypes. Nat Commun (2014) 5:3963. doi: 10.1038/ncomms4963 24999802 PMC4339277

[22] Ince N de la Monte SM Wands JR. Overexpression of human aspartyl (asparaginyl) beta-hydroxylase is associated with malignant transformation. Cancer Res (2000) 60:1261–6. 10728685

[23] Song K Xue X-p Wang W Huyan T Wang H Yang H. The distribution and expression profiles hydroxylasesome tumorous cell lines and tissues. Xi Bao Yu Fen Zi Mian Yi Xue Za Zhi (2010) 26:141–4. 20230674

[24] Yang H Song K Xue T Xue XP Huyan T Wang W. The distribution and expression profiles of human Aspartyl/Asparaginyl beta-hydroxylase in tumor cell lines and human tissues. Oncol Rep (2010) 24:1257–64. doi: 10.3892/or_00000980 20878118

[25] Jizong Z Hai D. The diagnostic significance of serum aspartate-asparagine β hydroxylase (ASPH) molecule combined with AFP and GP73 in the diagnosis of primary liver cancer. J Shandong University (2014) 52:78–80.

[26] Wang K Liu J Yan Z Li J Shi L Cong W. Overexpression of aspartyl-(asparaginyl)-β-hydroxylase in hepatocellular carcinoma is associated with worse surgical outcome. Hepatology (2010) 52:164–73. doi: 10.1002/hep.23650 20578260

[27] Zou Q Hou Y Wang H Wang K Xing X Xia Y. Hydroxylase activity of ASPH promotes hepatocellular carcinoma metastasis through epithelial-to-Mesenchymal transition pathway. Ebiomedicine (2018) 31:287–98. doi: 10.1016/j.ebiom.2018.05.004 PMC6013968 29764768

[28] Huang CK Iwagami Y Zou J Casulli S Lu S Nagaoka K. Aspartate beta-hydroxylasegiocarcinoma progression by modulating RB1 phosphorylation. Cancer Lett (2018) 429:1–10. doi: 10.1016/j.canlet.2018.04.041 29733964 PMC5985220

[29] Nagaoka K Bai X Ogawa K Dong X Zhang S Zhou Y. Anti-tumor activity of antibody-druggingte-β-hydroxylase in pancreatic ductal adenocarcinoma. Cancer Lett (2019) 449:87–98. doi: 10.1016/j.canlet.2019.02.006 30768955 PMC6411448

[30] Shimoda M Tomimaru Y Charpentier KP Safran H Carlson RI Wands J. Tumor progression-related transmembrane protein aspartate-beta-hydroxylase is a target for immunotherapy of hepatocellular carcinoma. J Hepatol (2012) 56:1129–35. doi: 10.1016/j.jhep.2011.12.016 PMC3328647 22245894

